# Harnessing the Synergy of Fe and Co with Carbon Nanofibers for Enhanced CO_2_ Hydrogenation Performance

**DOI:** 10.1021/acssuschemeng.3c05489

**Published:** 2024-01-18

**Authors:** Kevin Arizapana, John Schossig, Michael Wildy, Daniel Weber, Akash Gandotra, Sumedha Jayaraman, Wanying Wei, Kai Xu, Lei Yu, Amos M. Mugweru, Islam Mantawy, Cheng Zhang, Ping Lu

**Affiliations:** † Department of Chemistry and Biochemistry, Rowan University, Glassboro, New Jersey 08028, United States; ‡ Chemistry Department, Long Island University (Post), Brookville, New York 11548, United States; § Department of Civil and Environmental Engineering, Rowan University, Glassboro, New Jersey 08028, United States

**Keywords:** nanofiber catalysts, CO_2_ hydrogenation, iron, cobalt, electrospinning

## Abstract

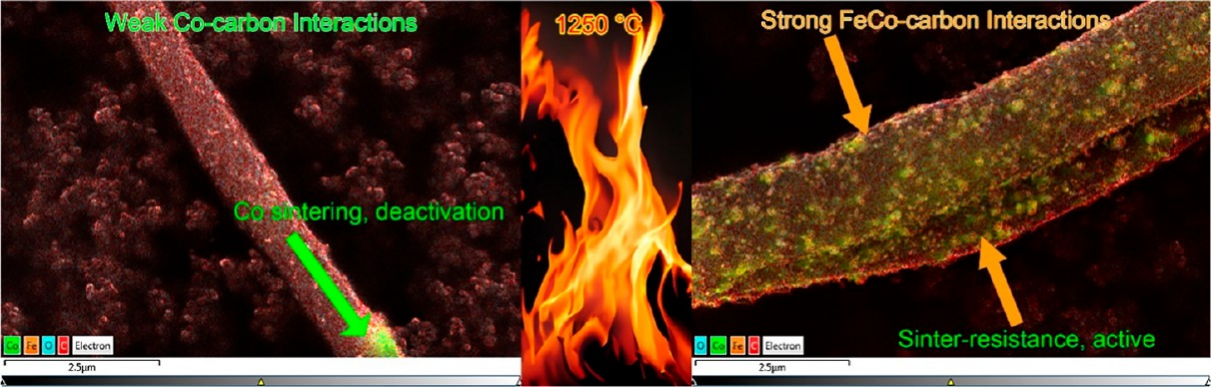

Amid growing concerns about climate change and energy sustainability, the need to create potent catalysts for the sequestration and conversion of CO_2_ to value-added chemicals is more critical than ever. This work describes the successful synthesis and profound potential of high-performance nanofiber catalysts, integrating earth-abundant iron (Fe) and cobalt (Co) as well as their alloy counterpart, FeCo, achieved through electrospinning and judicious thermal treatments. Systematic characterization using an array of advanced techniques, including SEM, TGA-DSC, ICP-MS, XRF, EDS, FTIR–ATR, XRD, and Raman spectroscopy, confirmed the integration and homogeneous distribution of Fe/Co elements in nanofibers and provided insights into their catalytic nuance. Impressively, the bimetallic FeCo nanofiber catalyst, thermally treated at 1050 °C, set a benchmark with an unparalleled CO_2_ conversion rate of 46.47% at atmospheric pressure and a consistent performance over a 55 h testing period at 500 °C. Additionally, this catalyst exhibited prowess in producing high-value hydrocarbons, comprising 8.01% of total products and a significant 31.37% of C_2+_ species. Our work offers a comprehensive and layered understanding of nanofiber catalysts, delving into their transformations, compositions, and structures under different calcination temperatures. The central themes of metal–carbon interactions, the potential advantages of bimetallic synergies, and the importance of structural defects all converge to define the catalytic performance of these nanofibers. These revelations not only deepen our understanding but also set the stage for future endeavors in designing advanced nanofiber catalysts with bespoke properties tailored for specific applications.

## Abstract 2

The dynamic synergy between Fe and Co, combined with their interaction with the carbon matrix, significantly enhanced the alloy’s electronic structure, catalytic activity, and robustness.

## Introduction

In the face of intensifying concerns over climate change and an impending energy crisis, catalysis stands out as an indispensable instrument in our global arsenal.^1^ The ever-increasing levels of carbon dioxide (CO_2_) in our atmosphere—a primary contributor to global warming—pose a dual-edged scenario: a formidable challenge and a potential opportunity.^2^ Catalysts are at the forefront of converting this greenhouse gas into valuable chemicals, offering the dual benefit of reducing greenhouse gas emissions and paving the way for energy-efficient chemical synthesis.^3^ Catalytic CO_2_ hydrogenation has gained significant attention as a credible scientific approach to counter these global challenges.^4,5^ This method transforms CO_2_ into useful energy fuels and chemicals, including carbon monoxide (CO), methane (CH_4_), and light olefins.^6^ Beyond addressing atmospheric CO_2_ accumulation, it heralds a path to a sustainable hydrocarbon-based energy future, promoting a more balanced global carbon footprint.^7,8^ Nevertheless, this vision is not without hurdles.^9^ The inherent thermodynamic stability of CO_2_ complicates its direct conversion to energy fuels and chemicals, particularly under milder pressure and temperature.^10^ One effective route to producing hydrocarbons from CO_2_ involves the reverse water–gas shift (RWGS) reaction, converting CO_2_ to CO, subsequently followed by Fischer–Tropsch synthesis (FTS) to yield long-chain hydrocarbons (RWGS-FTS).^11^ Currently, the research is heavily focused on enhancing the activity and selectivity of catalysts toward the desired products, primarily light olefins and higher hydrocarbons, which are key feedstocks for the chemical industry. Iron-based catalysts, attributed to their pronounced activity in both RWGS and FTS along with their abundance and low cost, stand out as the prime choice for CO_2_ hydrogenation.^12−14^ Historically, iron catalysts for CO_2_ hydrogenation have predominantly been supported on oxide or carbon substrates.^12^ However, iron-based catalysts supported on oxide materials showed generally low activity toward CO_2_ hydrogenation to light olefins.^15^ Traditional metal oxide supports, notably SiO_2_ and Al_2_O_3_, tend to form inactive compounds like silicates or aluminates post thermal treatment.^16^ Some oxides, such as the reducible TiO_2_, can obstruct the metal active sites because of the migration of mobile TiO_*x*_ under reducing conditions.^17^

Carbon materials stand out for their inherent stability in reductive atmospheres, resilience against water attack, and optimal metal–support interactions.^18^ These attributes ensure a high rate of iron reduction and carburization, making them favorable supports for iron-based FTS catalysts.^19^ A plethora of carbon materials, ranging from activated carbons, carbon nanotubes (CNTs), and carbon spheres to glassy carbon, carbon nanofibers (CNFs), and graphene, have been explored as supports for FTS catalysts.^20^ Typically, these carbon substrates modulate the synergy between the active phase and the supporting structure. Moreover, their chemical and thermal stabilities shine under harsh reaction conditions.^21^ Their intrinsic characteristics—like high-surface area, tuneable surface chemistry, and excellent reusability—enable them as support materials for FTS catalysts.^22^ One distinct advantage of carbon materials is their ability to act as reducing agents during thermal treatments while securing the metallic particles.^23^ This feature is crucial since the metallic phase often serves as the active sites for CO hydrogenation.^24^ Iron and cobalt catalysts anchored on carbon structures have demonstrated superior FTS activity when juxtaposed with their oxide-supported counterparts, resulting from the potential electron transfer between carbon and the metals.^25^

CNFs, distinguished by their robust chemical inertness and formidable mechanical strength, have emerged as promising carbon support materials in FTS applications.^26^ Notably, CNFs have become the choice support to explore the inherent size effects of iron or cobalt particles on FTS activity.^27,28^ de Jong et al. demonstrated that the Fe/CNF catalysts (containing 12% Fe) exhibited an impressive selectivity toward light olefins (52%) while substantially inhibiting CH_4_ production.^29^ This improved selectivity arose from the homogeneous distribution of Fe particles across the weakly interactive CNFs. Moreover, a noteworthy increase in the production of lower olefins was observed exclusively in the iron catalysts that were enhanced with Na and S on an inert carbon support. In contrast, the Fe/γ-Al_2_O_3_ catalyst demonstrated a pronounced selectivity toward methane production. This indicates that the inert CNF support facilitates the catalytic activity by enabling weak interactions with the iron particles. Furthermore, this catalyst’s performance can be further enhanced by the addition of Na and S. However, a weak metal–carbon bond strength might induce metal particle aggregation during reactions, leading to loss of active surface area and subsequent deactivation. Addressing this, our study ventured into synthesizing both monometallic and bimetallic Fe–Co catalysts encapsulated within CNFs by using the electrospinning technique. Thanks to its versatility, electrospinning employs electrohydrodynamic atomization to generate continuous nanofibers, subsequently forming 3D configurations with hierarchical porosity.^30^ Such configurations stem from the intentional alignment of nanofibers, facilitating a balanced dispersion of active metal precursors.^31−33^ Furthermore, electrospinning adepts in fine-tuning metal proportions in the resulting nanofiber catalysts.^34^ These resultant nanofibers boast a high-surface area, augmenting the accessibility to active catalytic sites,^35^ while their intrinsic high porosity enhances reactant and product diffusion rates.^36^

Different from conventional techniques of infusing metal ions into carbon materials, our approach interweaves carbon and metal precursors within nanofibers, later transforming them into their respective phases through thermal processing. This simple yet effective strategy significantly amplifies the metal–carbon synergy, rendering the couple resilient to harsh reaction conditions. Our results revealed the reinforced bond between Fe and carbon, underpinned by the formation of iron carbides. A systematical evaluation of their physicochemical attributes and catalytic performance in CO_2_ hydrogenation under ambient pressure showed that the bimetallic FeCo nanofiber catalysts (1/2 Fe/Co molar ratio)—subjected to a thermal treatment at 1050 °C—markedly excelled in catalytic activity and stability. These insights emphasize the promising horizon of FeCo nanofiber catalysts within supported catalysis, highlighting their instrumental role in addressing pressing environmental predicaments.

## Materials and Methods

### Reagents and Materials

Iron(III) acetylacetonate, an ACS reagent with a purity of at least 97.0% (Fe(C_5_H_7_O_2_)_3_), and cobalt(II) acetate tetrahydrate, an ACS reagent with a purity of at least 98.0% (Co(CH_3_COO)_2_·4H_2_O), were procured from Sigma-Aldrich. These compounds served as the precursors for Fe and Co, respectively. Polyacrylonitrile (PAN), having a weight-average molecular weight (Mw) of 150,000, was also sourced from Sigma-Aldrich and was used as the carbon precursor. *N*,*N*-Dimethylformamide (DMF), an anhydrous solvent with a purity of 99.9%, was obtained from VWR and used to dissolve both the metal salts and the PAN polymer, enabling the formulation of electrospinning solutions. To extract metals in catalysts to quantify the Fe and Co content, nitric acid (67–70%, ARISTAR PLUS grade for trace metal analysis, HNO_3_) and hydrochloric acid (34–37%, ARISTAR PLUS grade for trace metal analysis, HCl) were used, both of which were acquired from VWR. No additional purification was performed on the purchased chemicals. All water used in the experiments was purified using a Millipore Direct-Q 8 UV water purification system, resulting in a water resistivity of 18.2 MΩ·cm at a temperature of 25 °C.

### Synthesis of Monometallic Fe and Co and Bimetallic FeCo Nanofiber Catalysts

Nanofiber catalysts incorporating Fe and/or Co were synthesized through an electrospinning technique, using DMF solutions containing PAN, Fe(acac)_3_, and/or Co(OAc)_2_. This was followed by a nitrogen atmosphere pyrolysis process that facilitated the conversion of PAN and metal salt precursors to nanofiber catalyst structures. In a typical setup, the electrospinning solution was loaded into a 5 mL capacity syringe, which was fitted with a 22-gauge flat-ended metal needle of roughly 2.5 cm in length (BD Medical). The solution was extruded at a rate of 1 mL/h via a syringe pump (model Legato 110, KD Scientific) under 22 °C and a relative humidity of 45%. A DC power supply (model ES30P-5W, Gamma High Voltage Research) was used to apply a 15 kV voltage to the vertically aligned needle. This process generated a charged jet that elongated into ultrafine fibers, which were subsequently collected on a conductive receiving surface located approximately 15 cm beneath the needle tip. The obtained fibrous mat was subjected to a pyrolysis process with temperature stages at 450, 850, 1050, and 1250 °C under a nitrogen gas flow. Each stage was maintained for 1 h with a controlled temperature ramping rate of 10 °C/min. This process resulted in the transformation of PAN, Fe(acac)_3_, and Co(OAc)_2_ into carbon, Fe, and Co, respectively. After pyrolysis, the synthesized monometallic Fe and Co nanofiber catalysts, as well as the bimetallic FeCo nanofiber catalysts with 1/2 Fe/Co molar ratio, were allowed to dry in a vacuum oven maintained at ambient temperature for a duration of 24 h, prior to subsequent analyses and characterizations.

### Characterization

To assess the nanofiber morphology and structure before and after calcination, high-resolution field-emission scanning electron microscopy (SEM, Apreo model from FEI) was employed. To enhance electron conduction, samples were subjected to gold sputter-coating for a period ranging between 30 and 120 s, depending on the specific sample under study. Representative SEM images were obtained at a consistent working distance of 6 mm, employing an acceleration voltage of 10 kV and a beam current setting of 0.40 nA. For quantifying nanofiber dimensions, ImageJ software (developed by the NIH) was applied to the acquired SEM images. Subsequent statistical analysis of the fiber size distribution was performed using the OriginPro software package (OriginLab).

Atomic force microscopy (AFM) imaging of the nanofiber catalyst samples was conducted using a Bruker Dimension XR scanning probe microscope system (Santa Barbara, CA). For sample preparation, several drops of a nanofiber suspension in ethanol (concentration approximately 0.01%) were carefully placed onto a freshly cleaved mica surface (highest grade V1 mica discs, with a 12 mm diameter, sourced from Electron Microscopy Sciences) and allowed to dry completely. The AFM scans were conducted in the air, under ambient conditions of temperature and humidity. The tapping mode was used for these scans, employing OTESPA-R3 standard silicon probes (with tip radius <10 nm, spring constant = 26 N/m, and resonant frequency = 300 kHz) from Olympus Corp. Imaging was performed at a 1 Hz scanning rate, with a resolution set at 512 pixels ×512 pixels. For image processing, section analysis, and 3D simulations, NanoScope Analysis 3.00 software was utilized. From the AFM height images, average height values of the samples were determined.

To determine the chemical composition change of the nanofibers, infrared spectroscopy analyses were performed using a PerkinElmer Frontier spectrometer by employing the attenuated total reflection (ATR) method. The absorbance spectra for the nanofibers were recorded across a wavenumber range of 4000 to 650 cm^–1^, at a spectral resolution of 4 cm^–1^. For each sample, an average was taken from 128 scans.

A Rigaku ZSX Primus II X-ray fluorescence (XRF) spectrometer was deployed to assess the principal elemental constituents in the solid nanofiber catalyst samples. X-ray generation was achieved via a rhodium anode, operated at settings of 50 kV for voltage and an approximate current of 50 mA. In a typical procedure, approximately 0.1 g of the catalyst sample was positioned between two Prolene thin films (sourced from Chemplex Industries, Florida, USA) that were mounted on a tubular support. These prepared samples were then secured in circular stainless-steel cups of 40 mm diameter, which were fitted with 10 mm diameter polypropylene centering devices. Measurement procedures were conducted under a vacuum to enhance the data accuracy and reliability.

For Fe and Co quantification in nanofiber catalysts, an Agilent 7900 inductively coupled plasma mass spectrometer (ICP-MS) was employed. The initial step involved acid extraction of samples using concentrated HNO_3_ (67–70%), facilitated by periodic sonication over a 24 h period. Following this, the samples were filtered using a 0.45 μm syringe filter to remove carbon particles. The resulting clear filtrate was then diluted with a 1% HNO_3_ aqueous solution until the desired final concentrations, within the 1–200 ppb range, were attained. To establish a calibration standard, eight distinct solutions containing Fe and Co concentrations of 0, 1, 5, 10, 30, 50, 100, and 200 ppb were used. ICP-grade HNO_3_ (metal content <1 ppb) and HPLC-grade water (18.2 MΩ·cm at 25 °C, filtered through a 0.22 μm membrane filter) served as the solvent for preparing both sample and standard solutions.

Elemental distribution mapping in nanofiber catalysts was conducted via energy-dispersive X-ray spectroscopy (EDS) analysis. Calcined nanofiber catalysts without gold sputtering were analyzed. The operating conditions included a voltage of 15 kV (exceeding two times the Kα values for Fe and Co), a current of 1.6 nA, a working distance of 10 mm, and image magnifications of 10k/20k, resulting in an optimal dead time of approximately 30%.

The thermal degradation behavior of the as-spun composite nanofibers was characterized by using a TA SDT Q600 simultaneous TGA/DSC analyzer. Typically, about 10 mg of nanofiber samples in an alumina pan was heated in a controlled manner from room temperature (∼20 °C) to 1300 °C. The temperature was ramped at a consistent rate of 10 °C/min under a dry nitrogen atmosphere, with a purge flow rate set at 100 mL/min.

Diffraction data for the nanofiber samples were acquired by using a Bruker D8 Discover X-ray diffractometer. The system operated with a Cu Kα radiation source, set at a voltage of 40 kV and a current of 40 mA. The measurements were conducted with a step size of 0.02° and a dwell time of 0.5 s per step. The 2θ angle for these scans was varied between 5 and 90°.

Raman spectra were collected by using a Horiba LabRAM HR Evolution Raman spectrometer. A diode laser, with a wavelength (λ_ex_) of 532 nm, served as the excitation source for these analyses. In the setup for these experiments, the samples were positioned on a glass slide designed for the microscopic examination. A 50× objective lens was employed during these analyses. The spectra were recorded over a wavenumber range from 200 to 4000 cm^–1^. The laser, with a power output of 1.5 mW, was focused through a 50 μm slit, resulting in a focus spot size of approximately 1 μm^2^. For each spectrum, data were acquired in two separate 300 s exposures. To ensure comprehensive and representative sampling, data were collected from approximately nine distinct locations across the sample.

### Evaluation of the Catalytic Performance

The testing of nanofiber catalysts was conducted in a flow-bed reactor, comprising a quartz tube (inner diameter: 4 mm; outer diameter: 6.35 mm), operating at ambient pressure and varying temperatures. For each evaluation, around 100 mg of the catalyst, having a uniform mesh size in the range 40–60, was positioned within the quartz tube. Quartz wool was employed to securely encase the catalyst from both ends. Initially, the catalyst was subjected to a reduction treatment at 350 °C for 2 h under a flowing 50% H_2_/N_2_ stream (with a total flow rate of 40 mL/min). Following this, the catalyst was allowed to cool to a temperature of 275 °C, in preparation for CO_2_ hydrogenation. The reactor was fed with a gas mixture of CO_2_, H_2_, and N_2_ at atmospheric pressure, maintaining a volume ratio of 1/3/1 and a total flow rate of 40 mL/min. The temperature of the catalyst bed was progressively escalated from 275 to 500 °C in steps ranging from 25 to 50 °C. Real-time analysis of the effluent gas stream was performed by utilizing an Agilent 8890 gas chromatograph, equipped with both a flame ionization detector (FID) and a thermal conductive detector (TCD). For the separation and quantitative analysis of hydrocarbons, an HP-PLOT Q capillary column was interfaced with the FID. In contrast, a Mol Sieve 5 Å PLOT capillary column was employed in conjunction with the TCD for the detection and analysis of N_2_, H_2_, CO_2_, CO, and CH_4_. Automated sequential runs were set up to continuously monitor the catalyst performance at various temperatures, with six GC data points for each temperature set. The key parameters for evaluating the catalyst, such as CO_2_ conversion (eq 1), CO selectivity (eq 2), and hydrocarbon (CH_4_, C_2_–C_4_^0^, C_2_–C_4_^=^, C_5+_) distribution (eq 3), are formulated as indicated
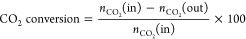
1
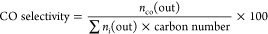
2
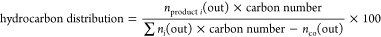
3*n*_CO_2__(in) is the initial molar quantity of CO_2_ fed into the reactor. *n*_CO_2__(out) is the molar quantity of unconverted CO_2_ exiting the reactor. *n*_product *i*_ is the moles of a given product *i*. Carbon number is carbon atoms contained in product *i*. Σ*n*_*i*_(out) is the cumulative molar quantity of carbon-containing products generated in the reaction.

## Results and Discussion

### Morphology of Monometallic (Fe and Co) and Bimetallic (FeCo) Nanofiber Catalysts

**Figure 1:**
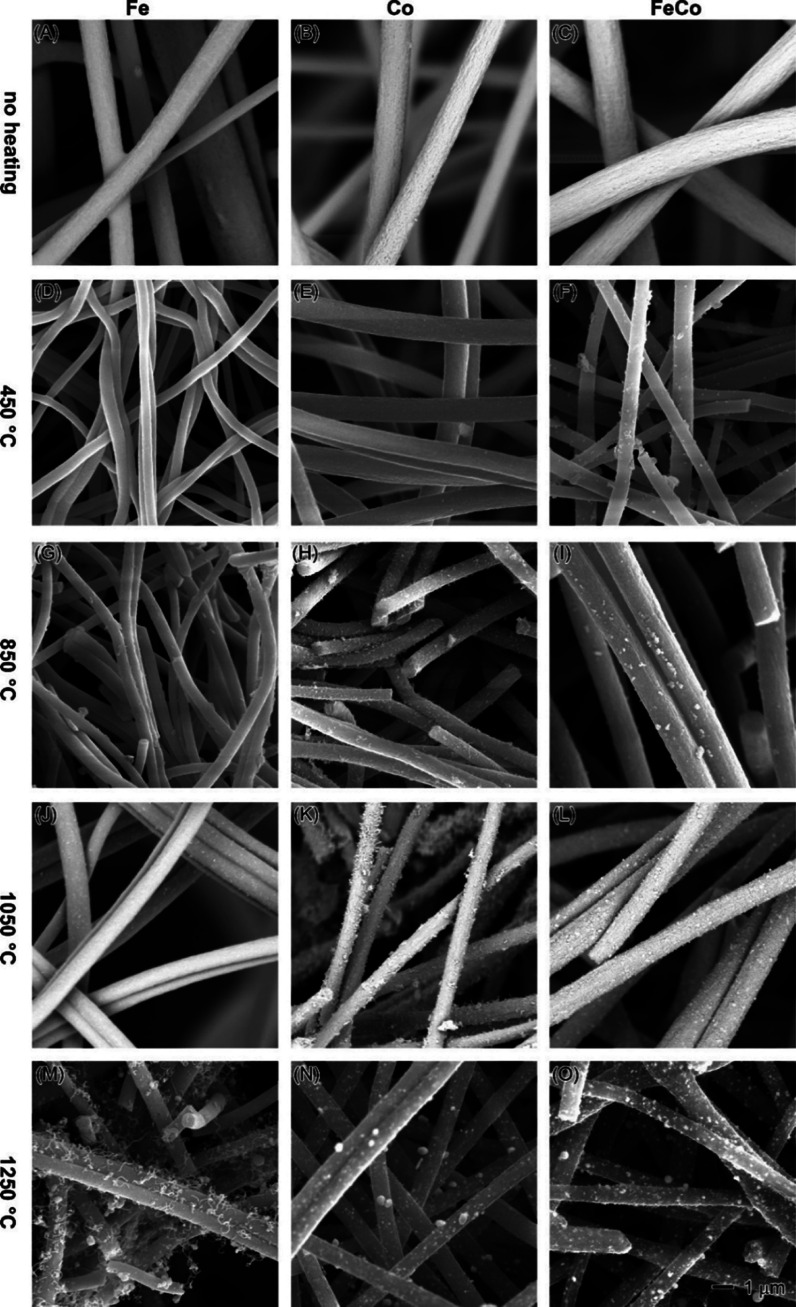
SEM images of Fe (the first column: A, D, G, J, and M), Co (the second column: B, E, H, K, and N), and FeCo (the third column: C, F, I, L, and O) nanofibers before (the first row) and after heating at 450 °C (the second row), 850 °C (the third row), 1050 °C (the fourth row), and 1250 °C (the fifth row). The 1 μm scale bar in (O) applies to all images.

Electrospinning was employed to fabricate precursor composite nanofibers that contained either monometallic Fe, monometallic Co, or a bimetallic blend of Fe and Co with a 1:2 molar ratio. The spinning solution was prepared by dissolving Fe(acac)_3_ (for Fe), Co(OAc)_2_ (for Co), and PAN (serving as the CNF precursor) in DMF. The metal precursor salts and PAN exhibited excellent solubility in DMF, resulting in uniformly tinted solutions with colors that varied based on the specific metal salt(s) employed. This observation is indicative of the complete dissolution of Fe^3+^ and Co^2+^ ions within the PAN solution, which is a factor critical to achieving homogeneity in the resulting electrospun nanofibers. As seen in Figure 1, the electrospinning operation was carried out smoothly, resulting in a nonwoven mat of uniform metal salt(s)-PAN composite nanofibers with dimensions of 20 × 20 × 0.3 cm. These nanofibers showed a consistent diameter throughout their lengths and were devoid of observable particles or irregularities, underscoring the uniform integration of metal salts within the polymer matrix. Following the fabrication step, these precursor composite nanofibers were subjected to a series of heat treatments under an inert atmosphere. The temperatures selected for these treatments, specifically 450, 850, 1050, and 1250 °C, have been consistently applied, as documented in our prior research.^37^ The goal of these treatments was to convert the Fe/Co salts to their metallic states and concurrently convert PAN to CNFs. Post-thermal treatment observations revealed an increase in sample brittleness, more pronounced in monometallic Fe or Co nanofibers compared to bimetallic FeCo nanofibers treated under identical conditions (Figure 1A–C). This difference could likely be attributed to the increased metal content and the resulting enhanced metal–support interactions in the bimetallic samples. Upon examination of the nanofibers post-450 °C treatment, the surfaces of the monometallic nanofibers appeared smooth and were devoid of visible metal particles (Figure 1D,E), while the bimetallic samples exhibited minor fragmentation (Figure 1F). When the treatment temperature was escalated to 850 °C, nanoparticles began to emerge on the nanofiber surfaces, as shown in Figure 1G–I. Notably, the monometallic Co nanofibers showcased a proliferation of nanofibrils, hinting at a potential formation of CNTs, as Co is a well-documented catalyst for CNT growth.^37,38^ With further elevation of the calcination temperature to 1050 °C, there was a notable increase in the number of nanoparticles on the nanofiber surfaces (Figure 1J–L). Impressively, numerous nanoparticles with dimensions of less than 10 nm persisted on the bimetallic FeCo nanofiber surfaces (Figure 1L). The final calcination step at 1250 °C resulted in the manifestation of larger, primarily 100 nm or greater, particles on the nanofiber surface, a consequence of extensive high-temperature metal sintering.

**Figure 2:**
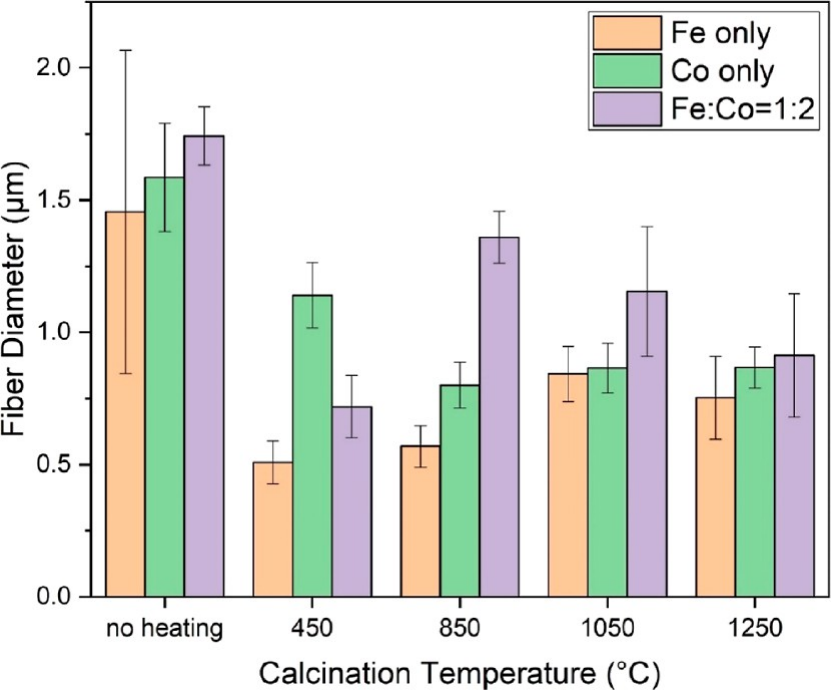
Average diameters of nanofiber catalysts with no heating and at 450, 850, 1050, and 1250 °C.

To gain a precise understanding of the transformations occurring in the nanofibers’ physical structure, we executed size determinations and statistical evaluations on a substantial selection of over 100 distinct fibers per sample, as illustrated in Figure 2. Prior to thermal treatment, the monometallic Fe and Co, as well as the bimetallic FeCo composite nanofibers, displayed average diameters of 1.455 ± 0.611, 1.586 ± 0.204, and 1.743 ± 0.111 μm, respectively, which is a direct consequence of the increased overall metal content in the fibers. Following heating to 450 °C, a notable shrinkage in size was recorded across all nanofiber types in comparison to their initial dimensions. Specifically, the reductions were 65.0% for Fe, 28.1% for Co, and 58.7% for the FeCo nanofibers. The pronounced contraction observed in the Fe-containing fibers may be reasonably attributed to the lower Fe content in Fe(acac)_3_ (15.81%), in contrast to the 23.66% Co content in Co(OAc)_2_. As the temperature increased to 850 °C, an intriguing trend emerged. While the monometallic Co nanofibers continued to shrink, exhibiting a 29.8% size reduction relative to their 450 °C counterparts, both monometallic Fe and bimetallic FeCo nanofibers experienced substantial size increases, peaking at 89%. This unexpected expansion in the Fe and FeCo nanofibers may be hypothesized as a potential interaction between Fe and the carbon matrix.

Transitioning to higher temperatures, the Co nanofibers remained relatively stable in size, sustaining an average diameter around 0.8 μm through the 1050 and 1250 °C treatments. In contrast, the Fe nanofibers displayed a slight size increase when treated at 1050 °C, followed by a decrease at 1250 °C—a pattern indicative of a sintering effect. Similarly, the bimetallic FeCo nanofibers experienced size reduction as the calcination temperature was advanced to 1050 and 1250 °C. SEM observations provide support for this trend, suggesting that the observed decreases in fiber dimensions could potentially result from nanoparticle formation on the nanofiber surfaces at these elevated temperatures.

**Figure 3:**
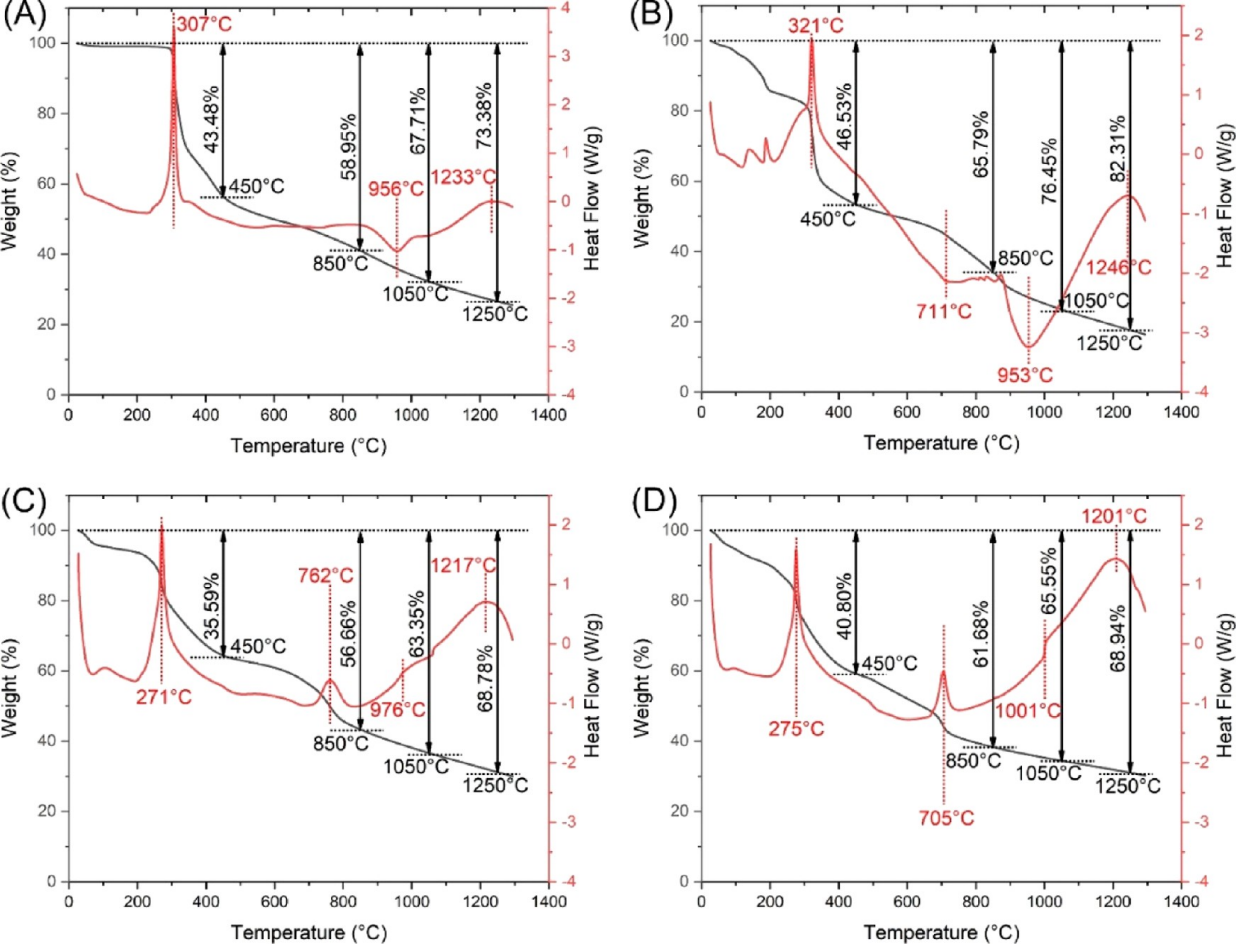
Simultaneous TGA-DSC thermograms showing thermal degradation profiles of (A) pristine PAN, (B) Fe-PAN, (C) Co-PAN, and (D) FeCo-PAN nanofibers from ambient temperature to 1300 °C in a dry nitrogen flow (100 mL/min).

Thermal degradation properties of both PAN and composite nanofibers were systematically assessed via synchronized TGA-DSC analysis. The results, illustrating the associated weight reduction and concurrent chemical reactions, are listed in Figure 3. In Figure 3A, the degradation trends of PAN are outlined. Up until 280 °C, PAN had a minimal weight loss of 1.2%, which is potentially indicative of the removal of the moisture content. Beyond 280 °C, three prominent peaks emerged in the PAN profile, which could be ascribed to dehydrogenation of organic components at 307 °C,^39^ carbonization to a carbon structure at 956 °C,^40^ and further graphitization toward graphitic structures at 1233 °C.^41^ During these transitions, noncarbon atoms were gradually eliminated, culminating in an aggregate weight loss of 73.38% at 1250 °C. Figure 3B captures the thermal degradation behavior of Fe-PAN composite nanofibers, and a notable divergence from pure PAN is observed. Specifically, the carbonization endothermic peak at 953 °C exhibited a significant amplification for the Fe-PAN composite nanofibers. This suggests the possibility of active interactions between Fe atoms and carbon within the structure. In stark contrast, as revealed in Figure 3C, this distinct endothermic peak was absent in the thermogram for the Co-PAN composite nanofibers, leading to the inference that Co may not be engaging in reactions with carbon under these conditions. Figure 3D, showing the degradation profile of FeCo-PAN composite nanofibers, exhibited an interesting shift in the baseline at 1001 °C. This behavior might be indicative of bonding interactions between the FeCo entities and carbon atoms. For all three composite nanofibers—but not in the thermogram of pure PAN—a peak in the 700–800 °C range was detected. This peak might be related to a transition in the metal state: an endothermic peak at 711 °C for Fe, an exothermic peak at 762 °C for Co, and an exothermic peak at 705 °C for FeCo. Furthermore, a continual decline in the mass of the composite nanofibers was observed. This dispelled the possibility that the observed enlargement in nanofiber diameters (Figures 1 and 2) could be ascribed to weight gain during the calcination process. Among all four samples analyzed, Fe-PAN emerged as the one that underwent the most substantial weight loss of 82.31% at 1250 °C. This pronounced degradation can likely be attributed to a series of complex interactions between carbon and iron, leading to the formation of iron carbide phases. The formation of these carbide phases typically involves the consumption of carbon, resulting in a notable decrease in the sample’s weight. On the other hand, both Co-PAN and FeCo-PAN showed relatively similar weight loss percentages at 1250 °C, clocking in at 68.78 and 68.94%, respectively. The close resemblance in their thermal degradation behavior is intriguing and may be explained by the dominant influence of the major component Co in both samples. The presence of a substantial amount of Co in the PAN matrix appeared to have markedly impeded the vaporization of carbon. This phenomenon is particularly noticeable with the Fe-PAN and FeCo-PAN samples. In the Fe-PAN sample, a significant formation of carbon fibrils was observed, likely resulting from carbon vaporization (Figure 1M). In contrast, the FeCo-PAN sample, despite containing an equivalent amount of Fe, did not exhibit the formation of carbon fibrils after calcination at 1250 °C (Figure 1O). This difference suggests that the addition of Co to the PAN matrix played a crucial role in stabilizing the carbon structure during the high-temperature treatment.

### Metal Content and Distribution in Nanofibers

The quantities of Fe and Co in the nanofibers were determined based on the theoretical percentages present in their precursor salts and considering the weight loss of PAN during its conversion to carbon materials. In earlier investigations, we observed a significant increase in the size of Fe particles when the metal loading exceeded 17%, leading to a detrimental effect on the catalytic activity.^42^ On the other end of the spectrum, metal loadings below 5% resulted in subpar hydrogenation performance, rendering the catalysts impractical for real-world applications.^43^ Additionally, our previous studies have indicated that a molar ratio of 1/2 (Fe/Co) is optimal for bimetallic nanofiber catalysts, promoting an enhanced electronic structure conducive to catalytic activity.^37^ Therefore, through recipe optimization, the Fe content was established at 5.00% and the Co content at 10.55% in the monometallic nanofibers (Table 1). To maintain identical metal loadings in the bimetallic nanofibers, the Fe and Co contents were set at 5.00 and 10.55%, respectively, adhering to a 1/2 Fe/Co molar ratio. Subsequent quantitative analysis, performed using ICP-MS, largely corroborated these figures. For the monometallic Fe nanofibers, which contained negligible Co, the average Fe content was measured at 4.83% (±0.27%), closely aligning with the theoretical value of 5.00%. Likewise, the Co monometallic nanofibers exhibited an average Co content of 9.67% (±0.48%), which closely approximates the theoretical expectation of 10.55%. In the bimetallic FeCo nanofibers, the measured Fe and Co contents were 5.34% (±0.32%) and 10.23% (±0.52%), respectively. XRF characterization further validated these ICP-MS measurements, indicating a 100:0 Fe/Co weight ratio in monometallic Fe nanofibers, a 0.24:99.76 Fe/Co ratio in monometallic Co nanofibers, and a 31.23:68.76 Fe/Co ratio in bimetallic FeCo nanofibers.

**Table 1:** Fe and Co Contents in Nanofiber Catalysts Determined by Theoretical Calculation/TGA and ICP–MS, and Fe/Co Weight Ratios Determined by XRF

samples	theoretical value/TGA		ICP-MS		XRF
	Fe (wt %)	Co (wt %)	Fe (wt %)	Co (wt %)	Fe/Co (wt/wt)
Fe	5.00	0	4.83 ± 0.27	0.026 ± 0.001	100:0
Co	0	10.55	0.16 ± 0.01	9.67 ± 0.48	0.24:99.76
FeCo	5.00	10.55	5.34 ± 0.32	10.23 ± 0.52	31.23:68.76

**Figure 4:**
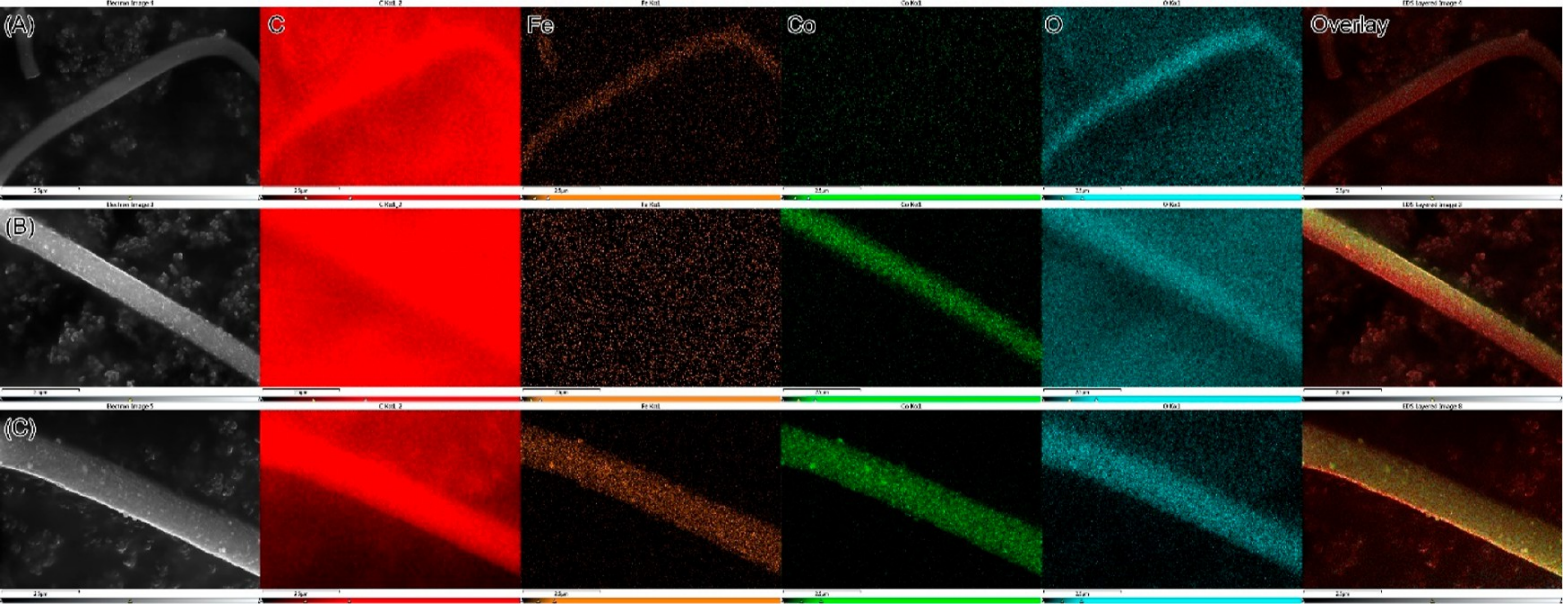
EDS mapping showing the distribution of elements in (A) Fe, (B) Co, and (C) FeCo nanofiber catalysts calcined at 850 °C.

The spatial distribution of elements, including Fe and Co, within both the monometallic and bimetallic nanofibers was assessed via EDS mapping. Figure 4 illustrates the distribution of four key elements—carbon (C), iron (Fe), cobalt (Co), and oxygen (O)—within the nanofibers. This figure also incorporates the relevant SEM images and corresponding overlays. Given that carbon constitutes the primary component of the nanofiber matrix and considering that conductive carbon tape was employed to secure the samples to the SEM sample stub holder, the carbon signal from the nanofibers was sometimes indistinguishable from the background carbon signal of the tape. The mapping showed that Fe and Co were uniformly distributed along the lengths of the monometallic and bimetallic nanofibers with no discernible regions of metal aggregation. It is important to note that since the samples were handled and transported in an oxygen-rich environment, adsorbed oxygen molecules were detected, resulting in recorded O signals.

### Catalytic Performance of Nanofiber Catalysts in CO_2_ Hydrogenation

The catalytic performance of the nanofiber catalysts was evaluated using a flow-bed reactor under atmospheric pressure. This deliberate choice is grounded in a strategic emphasis on sustainability and the aim of developing catalytic processes that are both efficient and environmentally conscious. Operating under atmospheric pressure offers a distinct advantage in terms of energy conservation as it circumvents the need for high-pressure equipment and the associated energy-intensive conditions required to maintain such environments.^44^ This is particularly pertinent given the global shift toward greener and more sustainable industrial practices. Moreover, evaluating catalytic performance at atmospheric pressure also provides a unique perspective on the intrinsic activity and selectivity of the catalysts, unobstructed by the potential influences of high pressure.^45^ It facilitates a more straightforward interpretation of the results, ensuring that the observed catalytic behaviors are predominantly attributed to the catalyst’s properties rather than external operating conditions.^46^ This is crucial for gaining deeper insights into the fundamental mechanisms driving the catalytic process and for guiding the future design and optimization of nanofiber catalysts.

Bimetallic FeCo nanofibers, designed with a 1/2 Fe/Co molar ratio, resulted in theoretical loadings of 5.00% Fe and 10.55% Co. For the sake of comparative analysis, monometallic Fe and Co nanofibers were introduced with identical loadings. All of these catalyst variants were subjected to a reduction and activation process at 350 °C for 2 h in a 50% H_2_ stream. This specific set of activation conditions was meticulously chosen based on insights garnered from our previous hydrogen temperature-programmed reduction (H_2_-TPR) studies.^42^ By implementing these conditions, we strategically facilitated the partial reduction of surface functional groups present on the carbon material, a step that plays a pivotal role in amplifying the catalytic activity of the system. This approach ensures a delicate balance, allowing us to enhance the catalyst’s functionality without pushing the iron species to a state of full reduction. Maintaining the iron species in their optimal oxidation state is of paramount importance as it directly correlates with achieving superior catalytic performance in the CO_2_ hydrogenation reactions. Throughout this phase, no hydrocarbons or other nonhydrocarbon byproducts were detected via online gas chromatography, thereby suggesting no underlying reactions between the carbon support and H_2_. Subsequent experiments on CO_2_ hydrogenation were conducted across a range of temperatures, each with a set of six GC injections, establishing a comprehensive data set for product analysis. It is noteworthy that CNFs, throughout these experiments, acted as inert references.

Diving deeper into the findings, Figure 7A illustrates a crucial observation: all nanofiber catalysts thermally treated at 450 °C showcased no measurable activity in terms of the CO_2_ hydrogenation. This absence of activity might have stemmed from the deposition of organic carbon materials on nanoparticle surfaces, ultimately inhibiting their active sites.^33^ However, a rise in the temperature to 850 °C marked a shift in this trend. All nanofibers exhibited enhanced catalytic activity, with the bimetallic FeCo nanofiber catalyst emerging as the most efficient, achieving an impressive CO_2_ conversion rate of 23.61%. This heightened efficiency could be attributed to the increased metal loading and the distinct presence of FeCo nanoparticles on the nanofiber surface (Figure 1I).^47^ The monometallic Fe and Co nanofiber catalysts registered CO_2_ conversion rates of 2.65 and 9.89%, respectively. However, even when combined, their total conversion remains lower than that of the bimetallic FeCo nanofiber catalyst, which have equivalent Fe and Co loadings. This underscores the synergistic advantage of combining Fe and Co in the CO_2_ hydrogenation. After being heated to 1050 °C, both the monometallic Fe and bimetallic FeCo nanofiber catalysts exhibited marked increases in activity, recording 26.43 and 46.47%, respectively. In contrast, the monometallic Co nanofiber catalyst’s CO_2_ conversion fell to a mere 1.21%, suggesting a deactivation likely due to thermal sintering (Figure 1K). This could be attributed to the weaker Co-carbon interactions compared with the Fe-carbon bonds. Even when accounting for the total metal loading, the bimetallic FeCo nanofiber catalysts outperformed the combined activity of the monometallic Fe and Co nanofiber catalysts under identical conditions. This serves as further evidence of the superior efficacy of the bimetallic catalysts over their monometallic counterparts.

**Figure 5:**
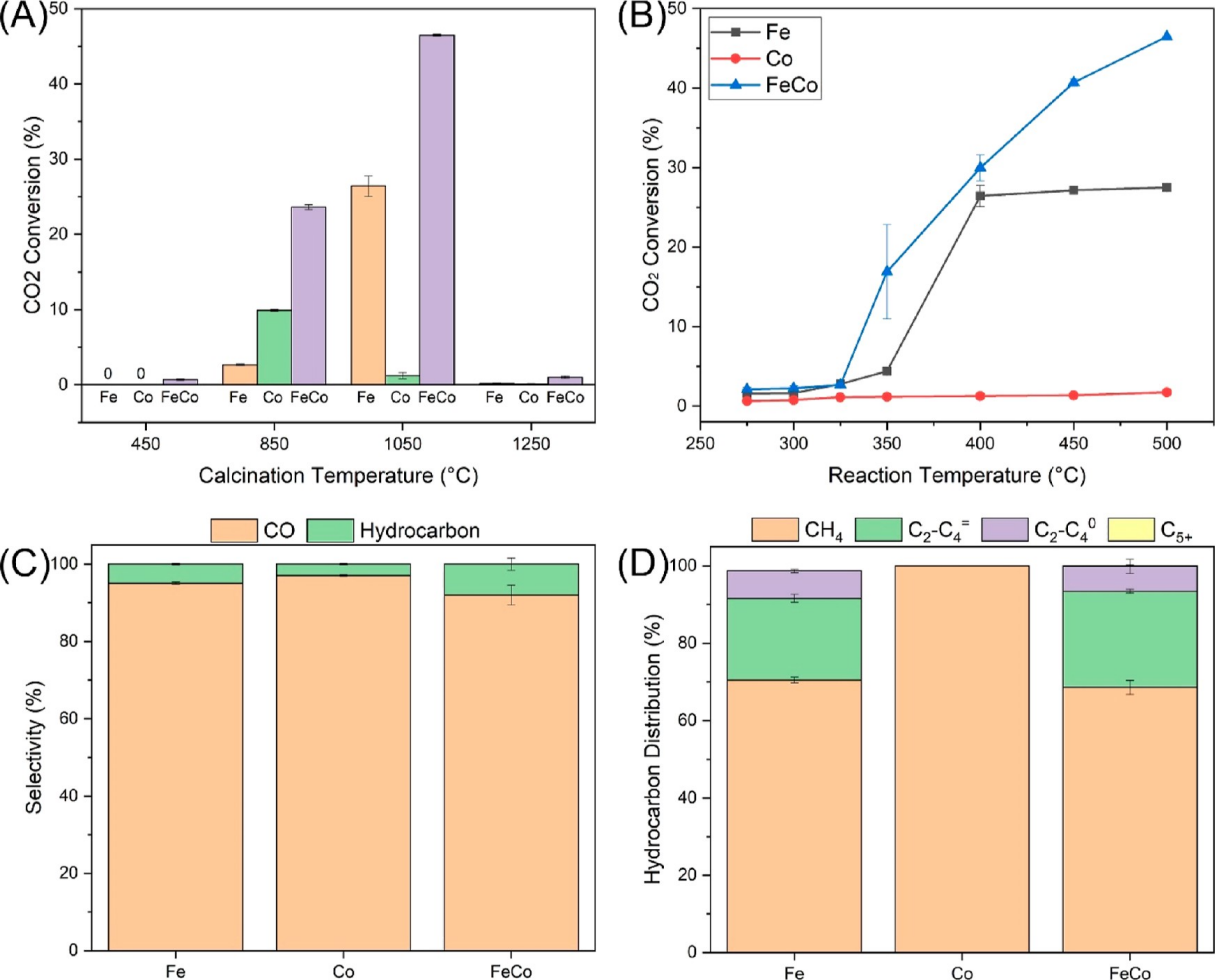
Catalytic performance of Fe, Co, and FeCo nanofiber catalysts. (A) Effect of calcination temperature used to prepare Fe–Co nanofiber catalysts on the CO_2_ conversion. (B) Effect of the hydrogenation reaction temperature on the CO_2_ conversion (calcination: 1050 °C). (C) CO and hydrocarbon selectivity (calcination: 1050 °C; reaction: 500 °C). (D) Hydrocarbon distribution (calcination: 1050 °C; reaction: 500 °C). Testing conditions including temperature: 275–500 °C, GHSV: 24,000 mL·g^–1^·h^–1^, H_2_/CO_2_ = 3:1, and P: atmospheric pressure.

**Figure 6:**
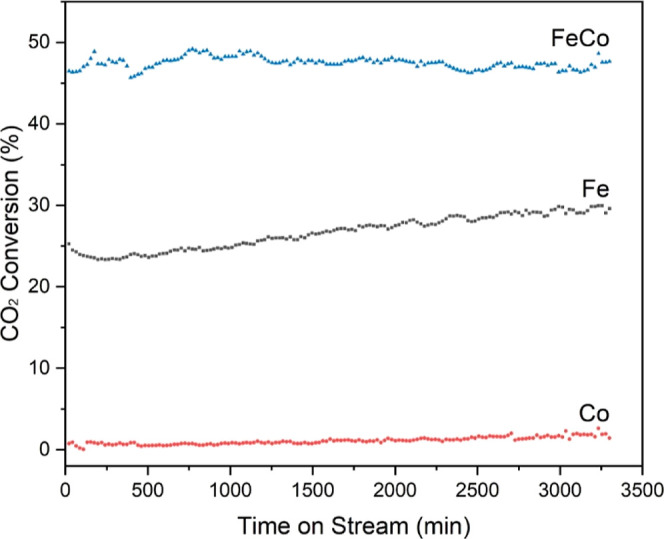
Stability of Fe, Co, and FeCo nanofiber catalysts calcined at 1050 °C. Testing conditions including temperature: 500 °C, GHSV: 24,000 mL·g^–1^·h^–1^, H_2_/CO_2_ = 3:1, and P: atmospheric pressure.

**Figure 7:**
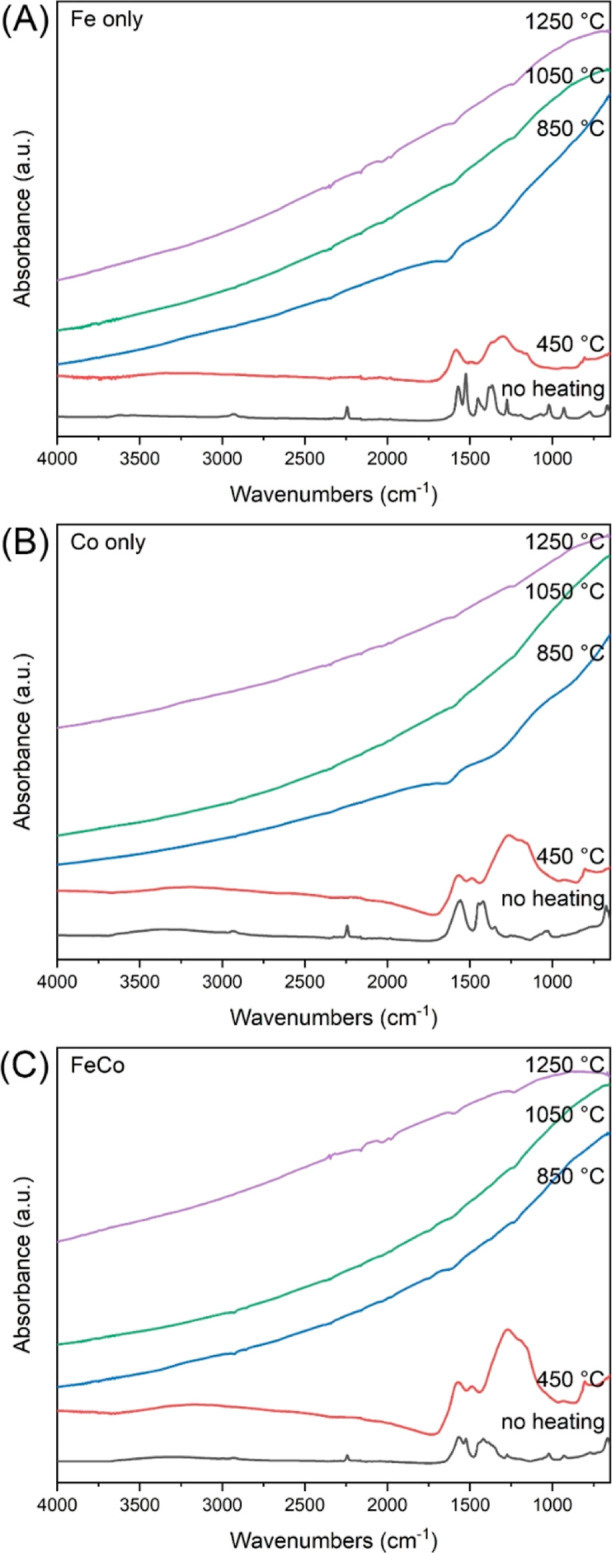
Infrared spectra of nanofiber catalysts before and after heating at different temperatures: (A) Fe only, (B) Co only, and (C) Fe/Co = 1:2.

As shown in Figure 5B, the level of CO_2_ conversion rises with increasing temperature, peaking at 500 °C. This suggests that the rate of CO_2_ conversion is more efficient at elevated temperatures. Notably, among the nanofiber catalysts preheated at 1050 °C, the monometallic Co catalysts consistently achieved around 1% CO_2_ conversion across all tested temperatures. In contrast, the CO_2_ conversion rate for the monometallic Fe nanofiber catalyst climbed from 1.58% at 275 °C to 26.43% at 400 °C, with only a marginal increase beyond this temperature up to 500 °C. The bimetallic FeCo nanofiber catalyst, on the other hand, increased its CO_2_ conversion from 2.08% at 275 °C to 29.97% at 400 °C. Remarkably, its activity surged by 55% at 500 °C compared to 400 °C, highlighting the superior thermal stability of the bimetallic catalyst. Figure 7C presents that the dominant product of CO_2_ hydrogenation is CO. The selectivity toward CO was 95.04% for Fe nanofiber catalyst, 97.04% for Co, and 91.99% for FeCo. It has been reported in the literature that selectivity toward CO can reach as high as 100% in the temperature range of 200–600 °C through the RWGS reaction, with CO_2_ conversion up to 50%.^48^ Significantly, of the three, the bimetallic FeCo nanofiber catalyst yielded the highest proportion of the more valuable hydrocarbons at 8.01%. Figure 5D shows the distribution of the hydrocarbon products. The monometallic Co nanofiber catalyst exclusively generated CH_4_. In contrast, catalysts containing Fe produced light olefins (C_2_–C_4_^=^) and light alkanes (C_2_–C_4_^0^). Notably, the bimetallic FeCo nanofiber catalyst produced a higher percentage of C_2+_ species (31.37%; 2.51% overall selectivity for 46.47% CO_2_ conversion) than the monometallic Fe nanofiber catalyst. The resultant C_2+_ species comprised 24.84% light olefins and 6.42% light alkanes. Moreover, only the bimetallic FeCo nanofiber catalyst gave rise to detectable C_5+_ species, albeit at a mere 0.17%. A portion of these C_2+_ species may be derived directly from the hydrogenation of CO_2_, bypassing the intermediate formation of CO.^49^ In terms of selectivity toward C_2+_ hydrocarbons, our catalysts exhibit modest performance. However, it is important to consider that the primary pathway in CO_2_ hydrogenation over the nanofiber catalysts is geared toward the production of CO. This is not a disadvantage per se as the production of CO is a valuable process in its own right, finding applications in various chemical industries.^50^ In comparison to similar studies in the literature, our catalysts stand out due to their exceptional thermal stability, high CO_2_ conversion, and CO selectivity at atmospheric pressure.^4,51^ While there are catalysts reported with higher selectivity toward C_2+_ hydrocarbons, these often operate under more stringent conditions, such as higher pressures, and may not exhibit the same level of thermal stability.^52^

The long-term stability of catalysts is a critical parameter for their practical application in industry. The nanofiber catalysts were subjected to rigorous stability testing under the conditions that had previously shown maximum activity for CO_2_ conversion. To ascertain their stability, these catalysts were maintained in a catalyst bed at 500 °C during CO_2_ hydrogenation, and their performance was monitored with regular intervals. Specifically, GC injections were executed every 22 min for a cumulative duration of 55 h (3300 min). The results, as depicted in Figure 6, elucidate the CO_2_ conversion trends for each of the nanofiber catalysts throughout the testing. Among the three, the monometallic Co nanofiber catalyst registered the lowest CO_2_ conversion, hovering around a mere 1%. This relatively low conversion could be attributed to weaker Co-carbon interactions, as hinted at in previous observations, or potentially to other deactivation mechanisms intrinsic to Co. On the other hand, the monometallic Fe nanofiber catalyst exhibited a significantly higher conversion. It initiated its performance at a promising 25.28% CO_2_ conversion. This rate then dipped to 23.33% before climbing again, reaching a peak of 29.60%. This fluctuating behavior suggests that there might be dynamic changes occurring on the catalyst surface or potential interactions with the reactants or products, leading to temporary deactivation, followed by reactivation. The standout performer was the bimetallic FeCo nanofiber catalyst. Its CO_2_ conversion rates consistently remained impressive, oscillating within a narrow range of 46–49%. Such a minimal fluctuation, coupled with its high conversion efficiency, underscores the bimetallic catalyst’s resilience and superior stability. The synergistic effect between Fe and Co in the bimetallic catalyst seems to enhance not only its initial activity but also its long-term stability. This performance is paramount in industrial applications, where catalysts are expected to function efficiently over extended periods without frequent replacement or regeneration.

### Impact of the Chemical Composition and the Crystalline Structure on the Catalytic Performance

The catalytic performance of nanofiber catalysts is intricately tied to their chemical composition and crystalline structure. To understand these interplays, the FTIR-ATR technique was employed to analyze the evolution of the chemical composition of these catalysts, both pre- and post-thermal treatments at varying temperatures. Figure 7 provides a comprehensive view, presenting the spectral signatures of the as-spun nanofibers and those subjected to treatments at 450, 850, 1050, and 1250 °C. All as-spun composite nanofibers displayed distinct absorptions tied to PAN, Fe(acac)_3_, and Co(OAc)_2_. Specific peaks at 2243 and 1662 cm^–1^ are indicative of the pronounced polarity of the nitrile group (C≡N stretching) inherent to PAN. Further characteristic absorptions for PAN are evidenced at 2936 cm^–1^ (attributed to alkyl C–H stretching) and 1452 cm^–1^ (corresponding to CH_2_ and CH_3_ bending).^53^ The 1568 cm^–1^ absorption uniquely pinpoints the carbonyl group’s presence in the acetylacetonate and acetate groups.^54^ Transitioning to the post-450 °C heating phase, a transformation is noted across all samples. The spectra manifested broad bands spanning from 1696 to 650 cm^–1^. Notably, the prior nitrile group vanished, yielding bands characteristic of C=N (1576 cm^–1^), C–C (1270 cm^–1^), and C=C (800 cm^–1^), a shift underpinned by intricate processes involving elimination, cyclization, and aromatization.^55^

Residual organic moieties present on the surfaces of the nanofiber catalysts can significantly hinder their catalytic performance. The presence of these organic moieties can obstruct the pathways leading to the active sites, thereby limiting the interaction between these active sites and reactant molecules. This phenomenon is akin to a blockage in a series of tunnels, preventing reactant molecules, in this case CO_2_, from reaching their desired destinations. For the two monometallic catalysts, this obstruction was so severe that the conversion rate for CO_2_ was virtually nonexistent, close to 0%. In the case of the bimetallic FeCo nanofiber catalyst, the conversion rate was marginally better but still negligible at 0.69%. Furthermore, the reactive sites on the catalyst are quintessential for the adsorption of reactant molecules and facilitate their subsequent transformation. If these sites were masked or blocked, the catalyst’s overall performance in driving the desired chemical reactions diminished. In essence, for a catalyst to exhibit optimal activity, not only is the nature and structure of the active site crucial but also its accessibility to reactants is equally imperative. The presence of organic residues serves as a reminder that the pretreatment or activation process for catalysts is of paramount importance. Properly cleaning or conditioning the catalyst surface can make the difference between an almost inactive catalyst and one that operates at the peak performance.

Upon subjecting the nanofiber catalysts to elevated temperatures of 850, 1050, and 1250 °C, notable changes in their sample spectra became evident (Figure 7). This shift in the spectral features resonates with the inherent properties of conductive carbon and metal structures. A crucial phenomenon observed in conducting solids is that the penetration depth of an electric field becomes shorter as the wavenumber increases, showcasing an inverse relationship.^56^ This observation suggested a critical transformation in the nanofiber catalysts’ constitution. The spectral changes were attributed to the successful conversion of the initial precursor compounds into their metallic forms or potentially into their oxide or carbide derivatives. Alongside this metal transformation, there was also the formation of graphitized CNFs, a structure known for their excellent conductivity and stability. An equally vital transformation was the complete elimination of any residual organic entities from the nanofibers as a result of the pyrolysis of PAN to carbon. Such a clean surface, devoid of obstructive organic residues, ensured that the catalyst’s reactive sites were unhindered and readily available. The accessibility of these sites was paramount for the catalyst’s functionality. The culmination of these transformations—metallic conversion, graphitization, and surface cleansing—ushered in a drastically enhanced catalytic performance. As illustrated in Figure 5, nanofiber catalysts calcined at temperatures of 850 and 1050 °C demonstrated a marked improvement in their ability to facilitate reactions, emphasizing the pivotal role that preparation conditions played in determining a catalyst’s performance.

**Figure 8:**
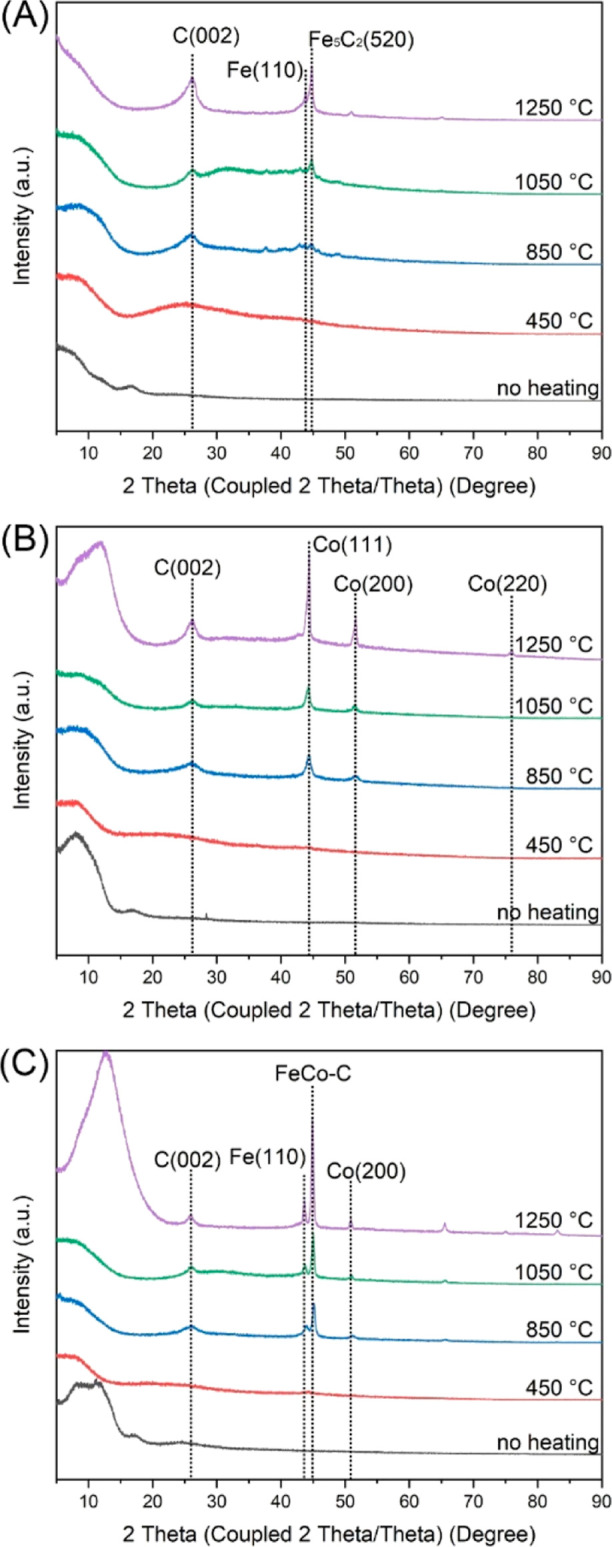
XRD patterns of nanofiber catalysts calcined at different temperatures: (A) Fe only, (B) Co only, and (C) FeCo (Fe/Co = 1:2).

To elucidate the transformation of PAN, Fe(acac)_3_, and Co(OAc)_2_ into carbon and metals, we analyzed the XRD patterns of nanofibers both before and after thermal treatments at varied temperatures. Figure 8 depicts these patterns for treatments at 450, 850, 1050, and 1250 °C. Initially, all untreated composite nanofibers exhibited a broad peak at around 16.4°, pointing to the amorphous structure of the PAN polymer. The XRD patterns barely exhibited specific peaks for Fe/Co salts at metal loadings below 10%. Nevertheless, with a higher Fe/Co loading (15%), distinct peaks became evident, notably at 2θ = 12.8°, representing the (011) plane of Co(OAc)_2_ (PDF 00-025-0372). Post the 450 °C thermal treatment, the amorphous PAN transformed into an amorphous carbon structure, which the XRD pattern highlighted with a broad peak at 25.1°.^57^ This transition also witnessed the emergence of a new peak at 44.4°, indicative of the (111) plane of the fcc Co structures (Figure 8B,C). Elevating the thermal treatment to 850 °C introduced two new peaks at 43.8 and 44.2°, correlating with the (110) plane of metallic Fe (PDF 00-006-0696) and the (510) plane of Fe_5_C_2_ (PDF 00-051-0997), respectively.^58^ Notably absent from the XRD diffractograms were Fe_2_O_3_ and Fe_3_O_4_ peaks, pointing to the exclusive formation of metallic Fe and its carbide during the calcination process. This pivotal observation—iron carbide’s formation—underscored a robust bond between Fe and the carbon support, which likely contributed to the enhanced CO_2_ conversion observed with both monometallic Fe and bimetallic FeCo nanofiber catalysts. We further noted that the intensity of the (110) Fe and (510) Fe_5_C_2_ peaks was amplified with rising temperatures, indicative of particle growth (Figure 8A). Concurrently, the increasingly distinct peak at 24.3° confirms the progressive graphitization of carbon beyond 850 °C, specifically representing the (002) plane of graphitized carbon.^42^

For the monometallic Co nanofiber catalyst, as displayed in Figure 8B, three prominent peaks appear at 44.3, 51.6, and 75.9°. These are associated with the (111), (200), and (220) planes of metallic Co, respectively.^59^ Importantly, no evidence of a Co carbide phase emerged from the diffractograms. One surprising observation is the absence of the CoO phase in both monometallic Co and bimetallic FeCo samples, despite various thermal treatments. A strong peak at 36.7° often associates with the (111) plane of CoO.^60^ Its absence suggests a nonformation of CoO. This could be attributed to the PAN or its thermal degradation products acting as reductants, facilitating the conversion of Co^2+^ (or Fe^3+^) to its metallic state. With PAN undergoing oxidation during its thermal decomposition, Co^2+^ (or Fe^3+^) played the role of oxidant, subsequently getting reduced.^61^Figure 8C presents the XRD patterns of bimetallic FeCo nanofiber catalysts. Peaks at 43.6 and 50.8° correspond to the (110) plane of Fe and the (200) plane of Co in their metallic forms, respectively. The strongest peak at 44.2° is assigned to the (510) plane of Fe_5_C_2_ because the (200) plane of Co and (110) plane of Fe are significantly weaker than their monometallic counterparts.^62,63^ This observation re-emphasized the robust Fe-carbon bonding in the Fe-containing samples, which not only resisted thermal sintering but also fostered robust metal–support interactions, thereby elevating the catalytic performance.^64^ In contrast, the monometallic Co nanofiber catalyst, lacking such effective Co-carbon interactions, became deactivated post 1050 °C calcination due to sintering (Figures 1K and 5B).^65^

**Figure 9:**
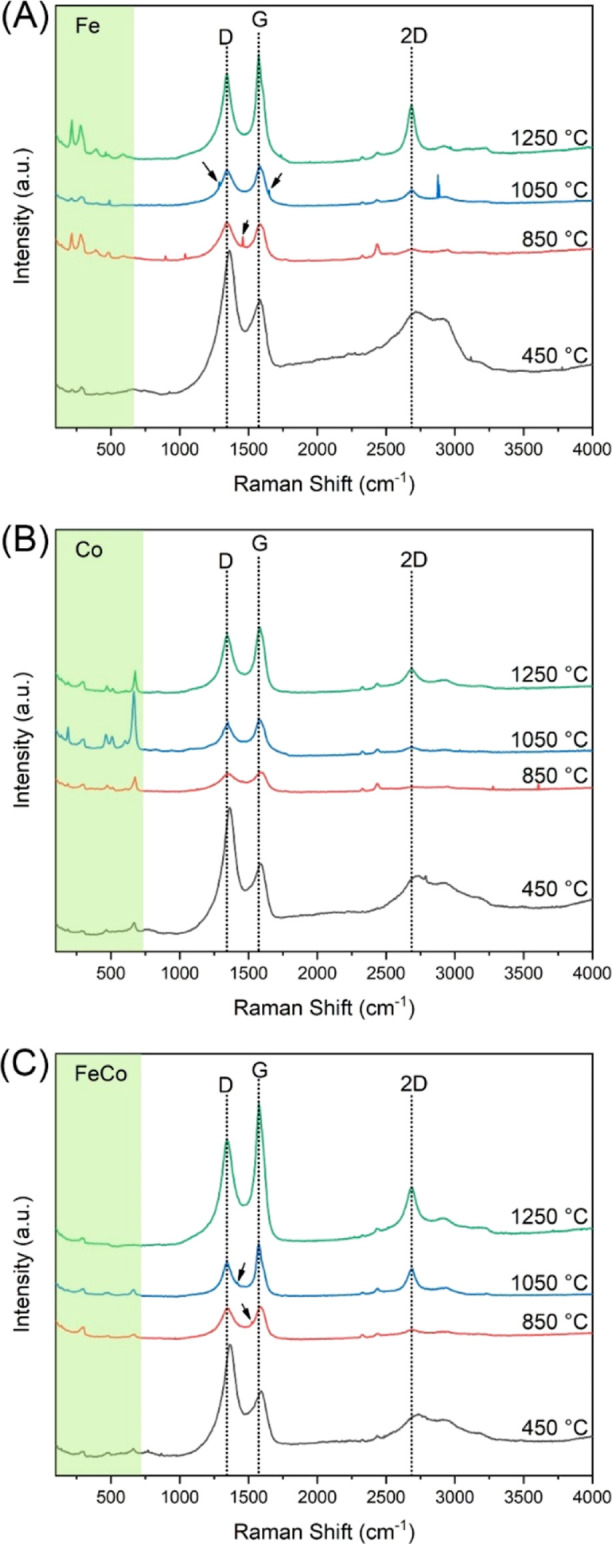
Raman spectra of (A) Fe, (B) Co, and (C) FeCo nanofiber catalysts heated at 450, 850, 1050, and 1250 °C.

The Raman spectra of the nanofiber catalysts subjected to various thermal treatments—450, 850, 1050, and 1250 °C—are presented in Figure 9. Within the spectral range 150–750 cm^–1^, we observed the vibrational frequencies associated with metal symmetric and asymmetric stretching. Such frequencies are heavily influenced by the nature of metal–metal and metal–ligand bonding.^66^ The intensification of the peaks within this range is attributed to the molecular excitation near the tail of their d–d transition.^67^ Specifically, the pronounced peaks at 216, 281, 393, 588, and 677 cm^–1^ are correlated to the stretching modes of Fe–Fe, Co–Co, and Fe–Co, complemented by heightened Fe–C interactions.^68^ Diving deeper into the carbon-related region of the spectra, two overarching peaks emerge at 1341 and 1580 cm^–1^, respectively, recognized as the D band and G band.^69^ The G band is indicative of the presence of sp^2^-hybridized carbon atoms, arising from the doubly degenerate E_2g_ symmetry at the center of the Brillouin zone. In contrast, the D band serves as a testament to the irregularities within the carbon lattice, attributed to resonant processes in proximity to the Brillouin zone boundary’s *K* point.^70^ Additionally, the presence of a 2D (or *G*′) band around 2685 cm^–1^ signifies the layering order of graphene sheets, derived from the scattering of two phonons, further indicating an in-plane transverse optical mode adjacent to the *K* point zone boundary.^71^

A notable trend was apparent across the spectra as the thermal treatment’s temperature was elevated. The diminishing intensity of the D band juxtaposed with the amplifying G band intensity across all samples confirms the increasing crystalline carbon regions within the nanofibers. Such a shift aligns with the graphitization of the nanofibers, a finding further substantiated by XRD (Figure 8). In tandem with this, the burgeoning intensity of the 2D band, particularly with the rise in temperature, underscores the amplifying graphene layering within these nanofibers. Despite these advancements in crystallinity, the persistent presence of the D band across all thermal treatments demonstrates the pervasive defects within the CNFs. Such defects, potentially manifesting as mesoporous channels, can be crucial in facilitating accessibility of reactive species to the catalyst’s active sites. Intriguingly, certain distinct peaks punctuate the D and G bands of both monometallic Fe (Figure 9A) and bimetallic FeCo (Figure 9C) nanofiber catalysts—peaks absent in the spectra of the monometallic Co (Figure 9B). These spectral nuances potentially point toward robust interactions between the Fe and carbon atoms in the nanofiber catalysts, suggesting unique structural or electronic interplays that may have catalytic implications.

**Figure 10:**
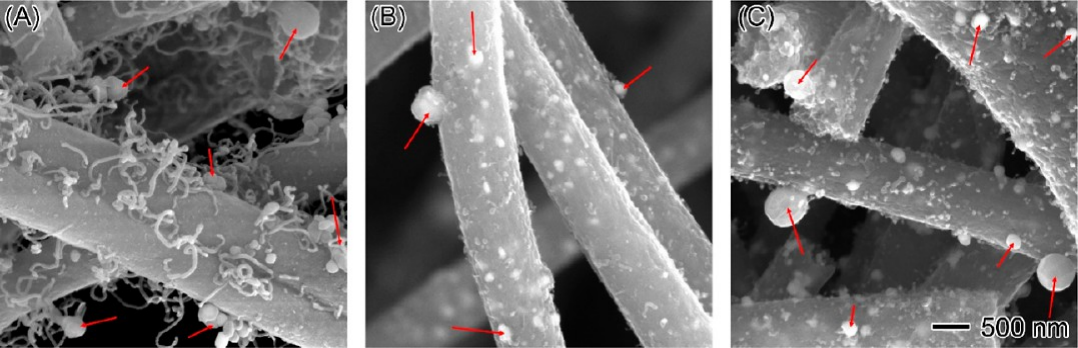
High-resolution SEM images showing the nanoparticle formation on (A) Fe, (B) Co, and (C) FeCo nanofiber catalysts at 1250 °C. The scale bar in (C) applies to all images.

**Figure 11:**
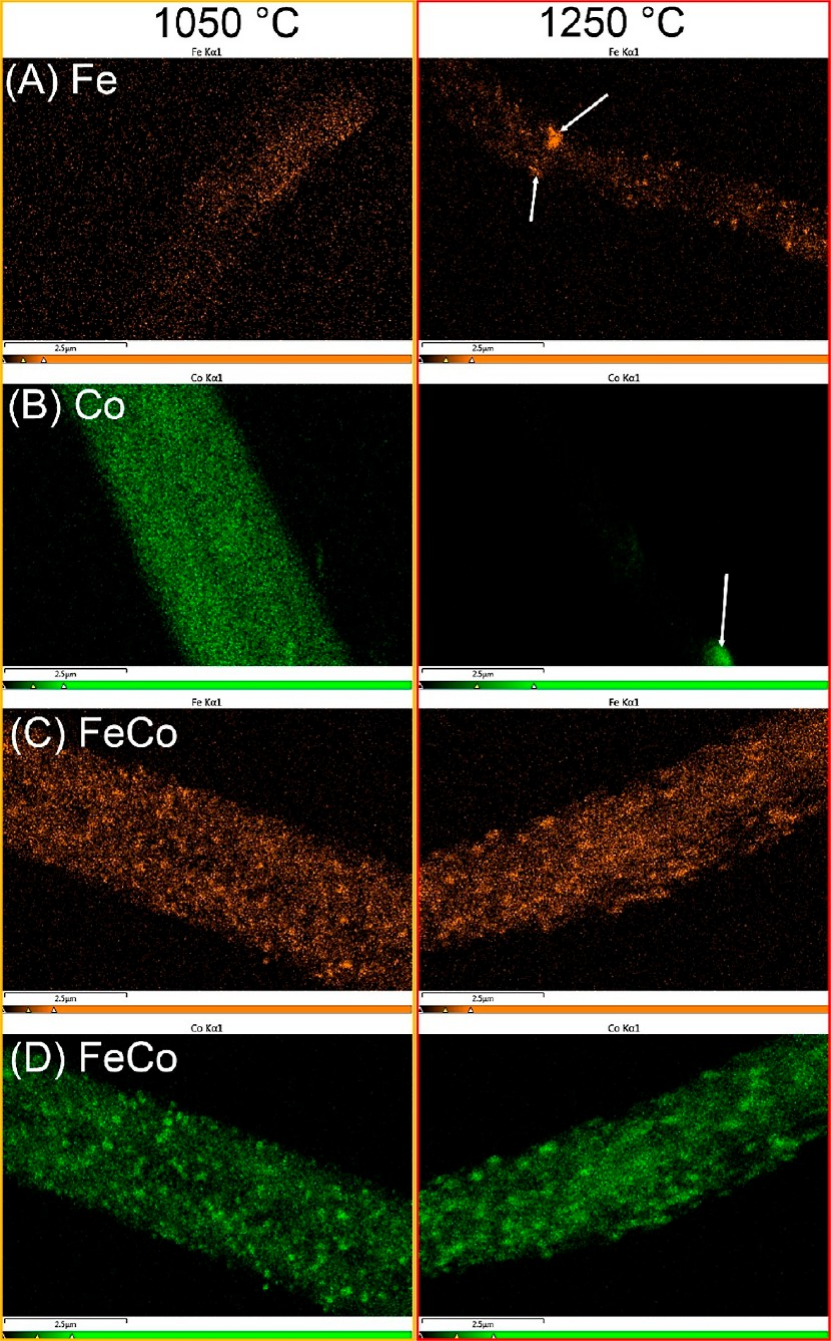
High-resolution EDS mapping showing the redistribution of Fe and Co in (A) Fe, (B) Co, and (C,D) FeCo under calcination from 1050 to 1250 °C.

Upon elevating the calcination temperature to 1250 °C, a discernible alteration in the morphology of the nanofiber catalysts was observed. Distinctly larger particles, ranging from 50 to 500 nm in diameter, appeared on the catalyst surface, as illustrated in Figure 10. To delve deeper into the composition of these particles and the overarching metal distribution on the nanofibers, high-resolution EDS mapping was undertaken. Notably, the phase transition exhibited the most pronounced differences between 1050 and 1250 °C. Figure 11A gives a lucid picture of the agglomeration of Fe into larger particles on monometallic Fe nanofibers. Yet, it is imperative to underscore that a substantial portion of Fe remained uniformly dispersed throughout the entire nanofiber structure. In stark contrast, the previously homogeneous distribution of Co in the monometallic Co nanofibers calcined at 1050 °C seemed to vanish in the EDS map of the 1250 °C-treated specimen. Here, only agglomerated Co particles could be discerned (Figure 11B). Such a holistic sintering of Co within the monometallic Co nanofibers hints at a more tenuous interaction between Co and carbon, especially when juxtaposed with the strong Fe–carbon interaction. The bimetallic FeCo nanofiber catalysts showcased superior resilience against sintering, as shown in Figure 11C,D. While the Fe/Co elements did show signs of agglomeration at elevated temperatures, the distribution of both metals across the nanofibers persisted, even when calcined at 1250 °C. Given the ostensibly weaker bond between Co and carbon, the preservation of Co throughout the nanofibers might be attributed to potential alloy formation between Co and Fe. Leveraging the potent interaction between Fe and carbon, the Co in the FeCo alloy remained firmly affixed to the carbon substrate, avoiding the all-encompassing sintering witnessed in its monometallic Co counterpart. The thermal stability of the catalysts often plays a pivotal role in shaping their catalytic performance. Accordingly, the Fe-enriched nanofiber catalysts manifested a markedly superior CO_2_ conversion rate when compared with the monometallic Co. Furthermore, the bimetallic FeCo nanofiber catalyst’s catalytic activity surpassed the cumulative activity of the separate monometallic Fe and Co catalysts. Such an enhancement can be ascribed to the synergistic effects stemming from the intimate interaction between Fe and Co as well as the refined electronic structure of the resultant alloy. This synergy often leads to improved catalytic activity due to the combination of properties, providing a cooperative effect that is often absent in monometallic systems.

**Figure 12:**
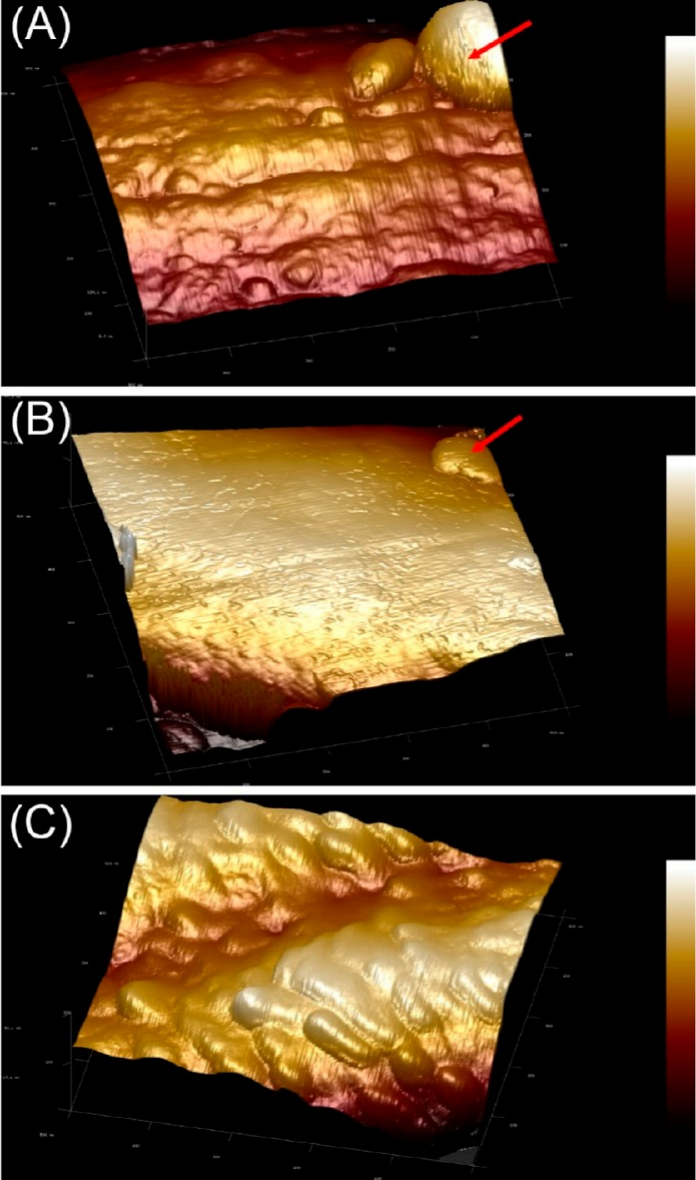
AFM images showing the formation of free particles on the surface of (A) Fe and (B) Co nanofiber catalysts. (C) Bound particles on the FeCo nanofiber catalysts.

A detailed investigation of nanoparticles on nanofiber catalysts was undertaken utilizing AFM scans, a powerful tool for achieving nanoscale resolutions. In this inspection, attention was narrowed to a single representative nanofiber with an approximate diameter of 1 μm, as shown in Figure 12. The reconstructed 3D images provide intricate insights into the surface topology and particle distribution. From these high-resolution images, Fe and FeCo domains manifested a rather uniform dispersion across the nanofiber surface, contrasting with the distinct, possibly sporadic, distribution of Co domains. The layout of these domains could indicate differences in metal–carbon affinities and interactions during the preparation and thermal treatment processes. The FeCo nanoparticles present a unique morphology; they are partially enveloped within the carbon matrix, leaving a fraction of their surfaces exposed. Such an architecture not only emphasizes a strong interfacial bond between the metal and carbon substrate but also offers an advantage from a catalytic perspective. The half-embedded nature ensures that while the metal particles are anchored and stabilized by the carbon matrix, their exposed regions remain available for catalytic interactions. This potentially enhanced active site accessibility, leading to improved catalytic performance, especially for reactions such as CO_2_ hydrogenation, where the interaction between the catalyst and reactant plays a pivotal role.

Furthermore, AFM scans unveiled the presence of larger, freestanding particles, predominantly exceeding 100 nm in size, on monometallic Fe and Co nanofibers. Their existence could be indicative of thermally induced sintering, wherein elevated temperatures lead to the coalescence of smaller particles into larger aggregates. This observation underscored the relatively low thermal resilience of the monometallic variants. Conversely, the absence of such aggregates on the bimetallic FeCo nanofibers accentuates their superior thermal stability. Such stability can be attributed to synergistic effects in bimetallic systems, where the metals can alloy or interact in a manner that impedes sintering, thereby preserving the particle size and distribution even at high calcination temperatures. AFM scans offer high-resolution insights into the nanofiber catalysts’ morphology and particle distribution, which are paramount for correlating structural attributes with catalytic performance and stability, guiding the design and optimization of future catalyst systems.

## Conclusions

In summary, this study offers a comprehensive understanding of the evolution and performance of both monometallic and bimetallic Fe–Co nanofiber catalysts under different calcination temperatures. As the temperature intensified, a distinct metamorphosis was witnessed: precursor materials transitioned into their respective metallic states, while also facilitating the emergence of graphitized CNFs. This transformation was meticulously traced through an array of techniques, including SEM, TGA-DSC, ICP–MS, XRF, EDS, FTIR-ATR, XRD, and Raman spectroscopy, which jointly mapped the conversion of PAN into a crystalline carbon structure, the maturation into metallic Fe/Co phases, and the formation of iron carbide compounds within nanofibers. Of the array tested, Fe-containing nanofiber catalysts stood out, with the bimetallic FeCo variant emerging as the most active catalyst for CO_2_ hydrogenation. Distinctly, the FeCo nanofiber catalyst calcined at 1050 °C exhibited an unparalleled CO_2_ conversion rate of 46.47%, demonstrating admirable stability (with fluctuations confined between 46 and 49%) across a 55 h testing period at 500 °C under atmospheric pressure. Furthermore, this catalyst was particularly adept at generating value-rich hydrocarbons, accounting for 8.01% of the output and a notable 31.37% of the C_2+_ species. The synergy between Fe and Co, together with their dynamic interactions with the carbon matrix, played a pivotal role in refining the electronic structure of the alloy, which, in turn, optimized catalytic activity and robustness. EDS mapping underscored the significance of FeCo alloying and its consequent bond with carbon, emphasizing the importance of even elemental distribution. The FeCo nanofiber catalysts showcased a remarkable resistance to sintering, even when exposed to a blistering 1250 °C—a resilience absent in their monometallic counterparts. This robustness was further spotlighted through AFM scans, where FeCo domains were discerned as being semiembedded in the carbon matrix—a revelation that underscores the potency of the metal–carbon bond, a bond poised to revolutionize the catalytic performance.
